# Borderline Personality Disorder With Depression Confers Significant Risk of Suicidal Behavior in Mood Disorder Patients—A Comparative Study

**DOI:** 10.3389/fpsyt.2020.00290

**Published:** 2020-04-17

**Authors:** John J. Söderholm, J. Lumikukka Socada, Tom Rosenström, Jesper Ekelund, Erkki T. Isometsä

**Affiliations:** ^1^ Department of Psychiatry, University of Helsinki and Helsinki University Central Hospital, Helsinki, Finland; ^2^ Department of Mental Disorders, Norwegian Institute of Public Health, Oslo, Norway; ^3^ Department of Psychology and Logopedics, University of Helsinki, Helsinki, Finland; ^4^ Department of Psyhiatry, University of Turku and Turku University Central Hospital, Turku, Finland

**Keywords:** suicide, suicide attempt, suicidal ideation, major depressive disorder, bipolar disorder, borderline personality disorder, Borderline Personality Disorder Severity Index, Columbia Suicide Severity Rating Scale

## Abstract

**Objective:**

We investigated risk factors for suicidal ideation and behavior among currently depressed patients with major depressive disorder (MDD), major depressive episode (MDE) in bipolar disorder (BD), or MDE with comorbid borderline personality disorder (MDE/BPD). We compared current and lifetime prevalence of suicidal ideation and behavior, and investigated dimensional measures of BPD or mixed affective features of the MDE as indicators of risk.

**Methods:**

Based on screening of 1,655 referrals, we recruited 124 psychiatric secondary care outpatients with MDE and stratified them into three subcohorts (MDD, BD, and MDE/BPD) using the Structured Clinical Interview for DSM-IV I and II. We examined suicidal ideation and behavior with the Columbia Suicide Severity Rating Scale (CSSRS). In addition, we quantified the severity of BPD symptoms and BD mixed features both categorically/diagnostically and dimensionally (using instruments such as the Borderline Personality Disorder Severity Index) in two time frames.

**Results:**

There were highly significant differences between the lifetime prevalences of suicide attempts between the subcohorts, with attempts reported by 16% of the MDD, 30% of the BD, and 60% of the BPD subcohort. Remarkably, the lifetime prevalence of suicide attempts in patients with comorbid BD and BPD exceeded 90%. The severity of BPD features was independently associated with risk of suicide attempts both lifetime and during the current MDE. It also associated in a dose-dependent manner with recent severity of ideation in both BPD and non-BPD patients. In multinominal logistic regression models, hopelessness was the most consistent independent risk factor for severe suicidal ideation in both time frames, whereas younger age and more severe BPD features were most consistently associated with suicide attempts.

**Conclusions:**

Among patients with major depressive episodes, diagnosis of bipolar disorder, or presence of comorbid borderline personality features both imply remarkably high risk of suicide attempts. Risk factors for suicidal ideation and suicidal acts overlap, but may not be identical. The estimated severity of borderline personality features seems to associate with history of suicidal behavior and current severity of suicidal ideation in dose-dependent fashion among all mood disorder patients. Therefore, reliable assessment of borderline features may advance the evaluation of suicide risk.

## Introduction

Patients suffering from mood disorders or borderline personality disorder (BPD) are at high risk of suicide. According to psychological autopsy studies, suicide is preceded by psychiatric disorders in the vast majority (c. 90%) of cases (1, 2). In long-term follow-up studies of patients with bipolar disorder (BD), major depressive disorder (MDD), and BPD, suicide mortality is significant (3, 4). Cumulative incidence of suicide among Danish psychiatric patients over 18 years was 4% for women and 7% for men with MDD, and 1% higher in BD (5). The risk of patients with softer BD features, such as periods of elevated mood of subsyndromal intensity or duration, remains unclear (6, 7). Previous suicide attempts (SAs) have been reported in up to half of BD and a third of MDD patients in psychiatric clinical samples (8). In a prospectively followed cohort of high-risk BPD patients, one third of the patients attempted suicide within 24 months (9). Identifying risk factors for suicidal behavior and ideation in these prevalent psychiatric disorders remains a research priority.

The suicidal process can be conceptualized as potentially progressing from ideation to attempt or death. The risk factors for different stages of suicidal ideation and acts may not be identical (10), and may differ across diagnostic and demographic groups (11), as well as temporally (9). Furthermore, effect of temporally distal risk factors may be mediated by more proximal clinical factors; e.g., influence of childhood maltreatment may be mediated by borderline personality features (12). Therefore, examining putative risk factors across diagnostic groups and stages of the suicidal process is important.

Established risk factors for suicide death in both MDD and BD include male gender and a family history of suicide (13–15), whereas risk factors common for SA include female gender, low age at onset, a recent depressive episode, comorbid anxiety, substance use, cluster B personality disorders, and a family history of suicide (14, 16). The risk of SA in mood disorder patients is related in a dose-dependent manner to time spent in depression, most significantly during major depressive episodes (MDE) (16, 17). Hopelessness is a risk factor for SA at least in MDD (18). Since BD patients have a lower age at onset and spend more time unwell than MDD patients, their lifetime risk for SA is higher (8, 11), and similar mechanisms seem to be at work in BPD (19). In a mixed sample of MDD and BD patients, features suggestive of at least a propensity towards BD were overrepresented among attempters (20). In BPD patients, worsening of comorbid MDD or substance use disorders (SUDs), childhood sexual abuse, traits of aggression and affective dysregulation, psychiatric hospitalization, being of a minority race, or having frequent changes in employment have been prospectively associated with a higher risk of SA (21–24).While BPD per se and certain BPD traits have been noted to be risk factors for SAs in depression, they are rarely dimensionally assessed, and thus, the importance of severity of BPD traits for risk of suicidal ideation and behavior remains unclear.

Although patients with MDD, BD, and BPD are at high risk of suicidal behavior, few studies have directly compared the prevalence and risk factors of suicidal ideation and attempts among these patient groups. Methodologically rigorous studies examining the association between adequately quantified BPD symptom/feature severity and suicidal ideation or behavior have been lacking, and the role of softer bipolar features in suicidal ideation and behavior is obscure. Such knowledge may help in identifying the most pertinent risk factors for suicidal behavior and ideation among mood disorder patients, thus helping in targeting treatment and preventive efforts.

The aim of this study was to investigate the prevalence and risk factors of SA among patients with MDE in MDD or BD, with or without comorbid BPD. Our *a priori* hypotheses were that (a) lifetime and recent SA are more prevalent and suicidal ideation more severe among BD and BPD/MDD patients than among MDD patients, (b) severe features or symptoms of BPD is a risk factor for suicidal ideation and behavior, and (c) mixed symptoms during depressive episodes are correlated with risk of SA or suicidal ideation.

## Materials and Methods

This was an observational cohort study conducted in Helsinki, Finland. Our sampling frame consisted of all patients referred with a possible diagnosis of affective disorder to the Northern and Eastern psychiatric outpatient clinics (offering secondary psychiatric care to a population of 234,415 adults).

The study was conducted according to the tenets of the Declaration of Helsinki (2013). Written informed consent was obtained from each participant. The research protocol was approved by the Ethics Committee of the Helsinki and Uusimaa Hospital District, and the research permit was granted by the City of Helsinki.

### Inclusion and Exclusion Criteria

Inclusion criteria were (1) current MDE, (2) a Montgomery-Åsberg Depression Rating Score (MADRS) ≥ 15, and (3) age 18–50 years. Exclusion criteria were (1) acute psychotic symptoms, (2) a diagnosis of schizophrenia or another primary psychotic disorder (not including MDD or BD with previous psychotic symptoms), (3) substance dependence (as in DSM-IV) with any current use, (4) ongoing use of large quantities of alcohol (24 units/week for men and 16 units/week for women) or benzodiazepine-type drugs ≥ 15 mg/day diazepam equivalents) in the last month, (5) any use of illicit drugs in the last month other than sporadic use of cannabis (once monthly in the last three months), (6) schizotypal or antisocial personality disorder, (7) a major neurocognitive or neurodevelopmental disorder, and (8) insufficient proficiency in the Finnish language to participate.

### Stratification and Recruitment

The patients were stratified into three principal diagnosis subcohorts: (1) MDD (no BPD), (2) BD, and (3) BPD with MDE. In case of comorbid BD and BPD, all BD type I patients were assigned to the BD subcohort, otherwise subcohort assignment was by principal clinical diagnosis.

Two of the authors (LS and JS) screened a total of 1,655 referrals for subjects. We preliminarily stratified these into three recruitment pools according to likely clinical diagnosis: (1) MDD with current MDE, (2) probable MDE in BD, and (3) probable MDE with comorbid BPD, and by gender. We aimed for equal representation in the strata, enriching currently underrepresented categories. If necessary, the patient to be interviewed was randomly selected within the stratum. Of screened referrals, 436 did not meet inclusion criteria, 855 were not contacted due to limited capacity or not reached, and 209 declined to participate. Thirty-one patients were excluded after a clinical interview, leaving a sample of 124 subjects. These were classified into the three final subcohorts according to final diagnosis.

Altogether, 31 patients were included in the BPD subcohort, 43 in the BD subcohort, and the remaining 50 in the MDD subcohort. Of these, 14 patients met the DSM criteria for both BD (type II) and BPD, eight of these were included in the BPD and six in the BD subcohort. Fourteen patients (six from the BPD, three from the BD, and five from the MDD subcohort) did not complete the Columbia Suicide Severity Rating Scale (CSSRS) (25) and are not included in the analyses of suicidal ideation and behavior. These patients did not differ from the main sample in MADRS scores or reported current suicidal thoughts or actions in the SCID.

### Interviews, Questionnaires, and Inter-Rater Reliability

All subjects were interviewed using the Structured Clinical Interview for DSM-IV-TR (SCID-I) (26) and SCID-II (27) (with the following personality disorders: paranoid, schizotypal, borderline, histrionic, narcissistic, avoidant, dependent, and obsessive-compulsive), MADRS (28), the Young Mania Rating Scale (YMRS) (29), Borderline Personality Disorder Severity Index (BPDSI) (30), part of the bipolar specifier (31), the Social and Occupational Functioning Assessment Scale (SOFAS) (32), and the CSSRS (25) in the use of which the performing authors (LS and JS) had received extensive training. Subjects were asked to complete questionnaires, including the Beck Depression Inventory-II (BDI-II) (33), the Patient Health Questionnaire-9 (PHQ-9) (34), the Overall Anxiety Severity and Impairment Scale (OASIS) (35), the McLean Screening Instrument for BPD (MSI-BPD) (36), the Perceived Social Support Scale (PSSS) (37), and the Beck Hopelessness Scale (HS) (38). We quantified possible mixed features of the index MDE *via* interview, scoring the occurrence of manic symptoms during the current MDE from 0 to 5 (Mix-MDE, see **Supplementary Material** for details). The subjects were met three times. Interrater reliability for principal diagnoses, tested by rating of videotaped interviews, was excellent (Cohen's κ for MDD 1.0, for bipolar disorder 0.898, for BPD 0.891).

As per the C-SSRS, we explored the severity of the subjects' most severe lifetime and recent suicidal ideation. This was quantified on the Suicidal Ideation Scale (SIS). Suicidal behavior included preparative acts in addition to aborted and interrupted and completed attempts (25). We classified our subjects into the following categories for both most severe lifetime and recent suicidali ideation: (1) no or mild suicidal ideation (SIS 0–2); (2) severe ideation (SIS 3–5) without SA, and (3) SA. For analyzing bipolarity dimensionally, we classified patients into the following ordinal categories: (1) no evidence of bipolarity, (2) bipolarity specifier positive but DSM-5 negative (subsyndromal hypomanias with a duration of 1–3 days only) (6, 7), (3) DSM-5 BD type II, and (4) DSM-5 BD type I. This ordinal measure was then used as a covariate in statistical analyses.

### Statistical Analyses

Data were assembled into a database using SQLite (http://www.sqlite.org/) and analyzed with IBM SPSS Statistics (https://www.ibm.com/analytics/us/en/technology/spss/) version 24. Chi-square test, Fisher's exact test, t-tests, and ANOVA were used in group comparisons. Furthermore, we analyzed correlates of suicidal ideation and behavior using multinomial logistic regression (using the latest available symptom severity scores), while subtracting suicide-related items from the MADRS, from the BPDSI, and from the number of positive BPD criteria in the SCID-II to avoid circularity.

Several multinomial logistic regression models were compared in assessing risk factors of lifetime and recent significant suicidal ideation and SA. Because each covariate has multiple regression coefficients in the multinomial model, overall significance of a covariate is tested by comparing models with and without it using a likelihood-ratio test. The covariates used in the initial lifetime model were gender, age, diagnosis of SUD, eating disorder, the bipolarity ordinal measure, most recent MADRS, HS, OASIS, number of positive BPD criteria (except suicidal ideation or behavior) in the SCID-II, PSSS, and duration of illness. In the initial model for recent suicidal ideation and behavior, the factors used were gender and diagnoses of eating or substance use disorders, while the covariates were the bipolarity ordinal measure, age, HS, OASIS, MADRS, BPDSI, Mix-MDE, PSSS, and duration of illness. Variables that were neither theoretically central nor statistically significant (i.e., for the lifetime model diagnosis of eating disorder or SUD, OASIS, PSSS and duration of illness; for the recent model diagnosis of eating disorder or SUD, OASIS, PSSS, duration of illness), were excluded from our final model.

## Results

No significant differences existed between the three patient subcohorts in gender, age, severity of depression, hopelessness, or level of functioning or perceived social support (**Table 1**). The severity of borderline symptoms was expectedly significantly higher in the BPD subcohort, and the YMRS scores lower in the MDD cohort, than in the others. Most (79%) of the patients in all subcohorts were treated with pharmacotherapy. However, they differed in use of mood stabilizers, used by 4%, 26%, and 10%; antipsychotics, used by 10%, 30%, and 16%; and antidepressants, used by 74% of the MDD, 56% of the BD, and 87% of the BPD subcohorts, respectively.

**Table 1:** Sociodemographic and clinical characteristics by subcohort.

			Subcohort												
			MDD			Bipolar Disorder			BPD			Total			
			Count	%/Mean	SD	Count	%/Mean	SD	Count	%/Mean	SD	Count	%/Mean	SD	Sig.
Sex	Male		21	42.0%		12	27.9%		8	25.8%		41	33.1%		0.237^a)^
	Female		29	58.0%		31	72.1%		23	74.2%		83	66.9%		
Age				31.4	10.2		31.6	9.1		28.5	7.6		30.8	9.2	0.301^b)^
Bipolar Disorder	Total		0	0.0%		43	100.0%		8	25.8%		51	41.1%		<0.001^a)^
		Type II	0	0.0%		33	76.7%		8	25.8%		41	33.1%		
		Type I	0	0.0%		10	23.3%		0	0.0%		10	8.1%		
BPD			0	0.0%		6	14.0%		31	100.0%		37	29.8%		<0.001^a)^
SUD			4	8.0%		19	44.2%		14	45.2%		37	29.8%		<0.001^a)^
Anxiety disorder			37	74.0%		34	79.1%		29	93.5%		100	80.6%		0.095^a)^
Eating disorder			10	20.0%		13	30.2%		10	32.3%		33	26.6%		0.405^a)^
MADRS				25.0	6.0		25.2	6.9		26.3	6.0		25.4	6.3	0.639^b)^
BDI-II				28.7	10.1		30.6	10.7		33.6	9.8		30.6	10.3	0.166^b)^
YMRS				1.7	2.7		3.4	3.7		3.5	2.6		2.8	3.2	0.007^b)^
OASIS				11.8	3.1		12.6	3.7		12.9	4.6		12.3	3.7	0.419^b)^
HS				12.5	3.9		11.8	4.8		10.9	3.9		11.9	4.2	0.287^b)^
PSSS				40.0	13.1		45.0	11.6		40.0	11.6		42.0	12.4	0.154^b)^
SOFAS				56.2	11.6		57.4	13.0		60.0	10.7		57.5	11.9	0.446^b)^
BPDSI				14.4	8.3		16.8	6.6		20.8	13.8		16.8	9.7	0.013^b)^

a) Pearson χ^2^.

b) One way ANOVA.

BDI2, Beck depression inventory 2; BPD, Borderline personality disorder; BPDSI, Borderline personality disorder severity index; HS, Hopelessness scale; MADRS, Montgomery Åsberg Depression Rating Scale; MDD, Major depressive disorder; PSSS, Perceived social support scale; SOFAS, Social and occupational functioning assessment scale; SUD, Substance use disorder; YMRS, Young mania rating scale.

Prevalence and severity of suicidal behavior and ideation is reported in **Table 2**. There were highly significant differences (p < 0.001) between the subcohorts in the lifetime prevalences of suicide attempts, suicidal behavior, and non-suicidal self-injury, all of which were most common in the BPD subcohort (in which they were reported by the majority of subjects). The median SIS for most severe lifetime suicidal ideation was 4 in the BPD subcohort and 3 in the others, and there were also significant differences between the subcohorts in the lifetime prevalence of significant suicidal ideation (p = 0.001), whereas the inter-subcohort differences in the prevalence of any suicidal ideation did not reach statistical significance (p = 0.066). During the preceding month the corresponding median SIS scores were 4 (the BPD subcohort) and 1 (others).

**Table 2:** Prevalence and severity of suicidal behavior and ideation.

		Subcohort												
		MDD			Bipolar Disorder			BPD			Total			
		Count	%/Mean	SD	Count	%/Mean	SD	Count	%/Mean	SD	Count	%/Mean	SD	Sig.
Lifetime	Attempts	7	15.6%	–	12	30.0%	–	15	60.0%	–	34	30.9%	–	<0.001^a)^
	Behaviour	12	26.7%	–	16	40.0%	–	20	80.0%	–	48	43.6%	–	<0.001^a)^
	Non-suicidal self injury	6	22.5%	–	9	22.5%	–	16	64.0%	–	31	28.2%	–	<0.001^a)^
	Ideation	39	86.7%	–	32	80.0%	–	25	100.0%	–	96	87.3%	–	0.066^a)^
	Significant ideation	31	68.9%	–	24	60.0%	–	25	100.0%	–	80	72.7%	–	0.001^a)^
	SIS		3.00	1.68		2.68	1.86		4.08	0.76		3.13	1.67	0.003^b)^
	SIIR		13.02	17.07		12.28	7.19		15.84	3.00		13.39	6.52	0.088^b)^
Recent	Attempts	3	6.7%	–	2	5.0%	–	5	20.0%	–	10	9.1%	–	0.138^c)^
	Behaviour	8	17.8%	–	4	10.0%	–	8	32.0%	–	20	18.2%	–	0.097^c)^
	Non-suicidal self injury	3	6.7%	–	6	15.0%	–	9	36.0%	–	18	16.4%	–	0.008^c)^
	Ideation	29	64.4%	–	21	52.5%	–	18	72.0%	–	68	61.8%	–	0.278^a)^
	Significant ideation	16	35.6%	–	11	27.5%	–	13	52.0%	–	40	36.4%	–	0.124^a)^
	SIS		1.76	1.80		1.25	1.48		2.04	1.65		1.64	1.67	0.147^b)^
	SIIR		8.13	7.20		6.90	7.08		9.08	5.97		7.90	6.89	0.447^b)^

a) Pearson χ^2^.

b) One-Way ANOVA.

c) Fisher's exact test.

BPD, Borderline personality disorder; MDD, Major depressive disorder; SIS, Suicidal ideation scale; SIIR, Suicidal ideation intensity rating.

**Figure 1:**
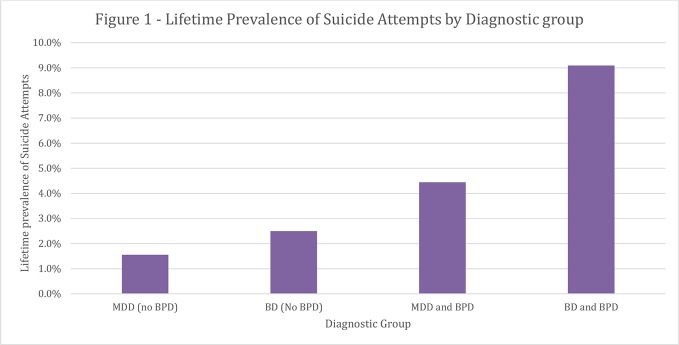
Lifetime prevalence of suicide attempts by diagnostic group.

When grouping patients purely into four groups by MDD/BD and BPD-diagnosis status (instead of by subcohorts) there were significant differences in the lifetime prevalences of SA (Fisher's exact test, p < 0.001), with earlier SA reported by 15.6% of MDD patients without comorbid BPD, 25.0% of BD patients without BPD, 44.4% of MDD patients with comorbid BPD, and 90.9% (10 out of 11) among BD patients with comorbid BPD (**Figure 1**).

The multinominal regression model for lifetime suicidal behavior and ideation explained these phenomena with high statistical significance (2LL = 184.013; χ2 (12) = 42.635; p < 0.001), and a moderate effect size (Nagelkerke pseudo-R2 = 0.368, **Table 3**). The covariates that significantly contributed to the model were age (p= 0.040), MADRS (p = 0.003), HS (p = 0.019), and number of positive BPD criteria (p = 0.004), but not the bipolarity measure (p = 0.346) or gender (p = 0.151).

**Table 3:** Most severe lifetime suicidal ideation/behavior - Multinominal logistic regression models.

Suicidality^a)^		Uncontrolled					Multiple (adjusted) regression				
		B	OR^b)^	95% CI		Sig.	B	OR^b)^	95% CI		Sig.
Significant ideation, no attempt	Age	-0.032	0.969	0.914	1.027	0.291	-0.043	0.957	0.891	1.028	0.234
	Bipolarity Ordinal	-0.360	0.698	0.419	1.161	0.166	-0.399	0.671	0.389	1.157	0.151
	MADRS	-0.008	0.992	0.894	1.100	0.874	0.043	1.044	0.932	1.170	0.455
	HS	0.105	1.111	0.987	1.249	0.080	0.140	1.150	1.002	1.321	0.047
	Nr of pos. BPD criteria	0.045	1.046	0.974	1.124	0.218	0.268	1.307	0.943	1.813	0.108
	Sex (male)	1.190	3.286	1.025	10.528	0.045	1.223	3.396	0.957	12.051	0.059
Attempt	Age	-0.052	0.949	0.903	0.997	0.016	-0.081	0.922	0.864	0.985	0.018
	Bipolarity Ordinal	-0.137	0.872	0.584	1.303	0.396	-0.213	0.808	0.495	1.321	0.443
	MADRS	0.090	1.094	1.005	1.192	0.003	0.154	1.166	1.053	1.291	0.003
	HS	0.125	1.133	1.027	1.252	0.009	0.170	1.185	1.044	1.345	0.009
	Nr of pos. BPD criteria	0.080	1.083	1.018	1.152	0.003	0.461	1.586	1.172	2.146	0.001
	Sex (male)	0.602	1.825	0.667	4.998	0.201	0.786	2.194	0.658	7.312	0.190

The model explained suicidality statistically significantly (-2LL = 184.013; χ2 (12) = 42.635; p < 0.001) with a moderate effect size (Nagelkerke pseudo-R2 = 0.368). The model fit was good and resulted in statistically similar predicted and observed suicidality distributions (Pearson and deviance statistic yielded p = 0.185 and p = 0.862, respectively).

a) The reference category is: No or mild ideation (SIS 0-2).

b) OR equals Exp(B).

BPD, Borderline personality disorder; HS, Hopelessness scale; MADRS, Montogomery Åsberg Depression Rating Scale; OR, Odds ratio; SIS, Suicidal ideation scale.

The recent suicidal behavior and ideation model was also highly significant (-2LL = 149.186; χ2 (14) = 44.670; p < 0.001), and the effect size moderate (Nagelkerke pseudo-R2 = 0.403, **Table 4**). Age (p = 0.009), HS (p = 0.010), MADRS (p = 0.013), and BPDSI (p < 0.001) were significant contributors to this overall model, while gender (p=0.552), the bipolarity ordinal measure (p = 0.984), and Mix-MDE (p = 0.502) were not.

**Table 4:** Recent suicidal idation/behavior - multinominal logistic regression models.

		Uncontrolled					Controlled				
		B	OR^b)^	95% CI		Sig.	B	OR^b)^	95% CI		Sig.
Significant ideation, no attempt	Bipolarity Ordinal	-0.076	0.927	0.631	1.360	0.697	0.045	1.046	0.643	1.701	0.856
	Age	-0.029	0.971	0.926	1.018	0.226	-0.082	0.921	0.862	0.984	0.015
	HS	0.137	1.147	1.038	1.267	0.007	0.181	1.199	1.058	1.358	0.004
	MADRS	0.130	1.139	1.051	1.235	0.002	0.135	1.145	1.038	1.263	0.007
	BPDSI	0.103	1.108	1.044	1.177	0.001	0.134	1.144	1.057	1.237	0.001
	Mix-MDE	0.002	1.002	0.945	1.062	0.950	-0.050	0.951	0.869	1.041	0.274
	Male sex	0.218	1.243	0.525	2.942	0.620	0.612	1.844	0.633	5.375	0.262
Attempt	Bipolarity Ordinal	-0.110	0.896	0.482	1.663	0.727	0.026	1.026	0.486	2.167	0.947
	Age	-0.086	0.918	0.836	1.008	0.073	-0.112	0.894	0.804	0.994	0.039
	HS	0.010	1.010	0.877	1.164	0.885	0.090	1.094	0.928	1.290	0.286
	MADRS	0.089	1.093	0.970	1.232	0.143	0.110	1.116	0.979	1.274	0.101
	BPDSI	0.125	1.133	1.038	1.237	0.005	0.136	1.146	1.029	1.277	0.013
	Mix-MDE	0.029	1.029	0.945	1.121	0.507	-0.042	0.959	0.846	1.087	0.514
	Male sex	-0.199	0.820	0.194	3.472	0.787	0.174	1.190	0.241	5.870	0.831

The model explained suicidality statistically significantly (-2LL = 149.186; χ2 (14) = 44.670; p < 0.001), and a moderate effect size (Nagelkerke pseudo-R^2^ = 0.403). The model fit was good and resulted in statistically similar predicted and observed suicidality distributions(Pearson p = 0.324, deviance p = 0.999).

a) The reference category is: No or mild ideation (SIS 0-2).

b) OR equals Exp(B).

BPDSI, Borderline personality disorder severity index; HS, Hopelessness scale; MADRS, Montogomery Åsberg Depression Rating Scale; Mix-MDE, Mixed symptoms during major depressive episode; OR, Odds ratio; SIS, Suicidal ideation scale.

A significant correlation existed between severity of recent suicidal ideation and BPDSI scores (Pearson correlation: 0.383, p < 0.001), which remained significant both among BPD (Pearson correlation: 0.586, p < 0.001) and non-BPD patients (Pearson correlation: 0.278, p = 0.012).

As a post-hoc analysis, we replaced the total BPDSI score in the multinomial regression model for recent suicidal behavior and ideation with the BPDSI subscores (except the self-injury category). The model was significant (p > 0.001). Of the BPDSI subcategories, sensitivity to abandonment (p = 0.004, OR = 2.070, 95% CI [1.264 - 3.391]) and anger/rage conferred increased odds of having made an attempt (p = 0.007, OR = 2.719, 95% CI [1.318 - 5.611]); the others did not reach statistical significance.

## Discussion

In this observational clinical study of treatment-seeking major depressive patients, we investigated the prevalence of and risk factors for suicidal ideation and attempts. We compared depressed patients with MDD, BD, and/or BPD. As hypothesized, we found marked differences in the lifetime prevalence of suicide attempts, which was twofold in the BD and fourfold in the BPD/MDE subcohort compared with MDD patients. Severity of BPD features was also strongly associated with suicidal ideation, both recently and over the lifetime. However, we did not find mixed symptoms during the index MDE to be a risk factor for suicidal behavior and ideation in this cross-sectional and retrospective analysis.

### Strengths and Weaknesses

Strengths of this study include the comparison of three clinically and epidemiologically central diagnostic groups with a high suicide risk, the representativeness of recruitment by stratified sampling, the standardized and rigorous assessment of dimensional aspects of psychiatric pathology such as BPD and suicidal ideation and behavior with the BPDSI and the CSSRS, the excellent inter-rater reliability, and the analysis of our results in two time frames (current and lifetime). Our main limitations include moderate sample size, retrospective evaluations, and unknown inter-rater reliability of the BPDSI. Features of our study relevant for interpretation and generalizability of the findings include the observational design, i.e., treatments provided were not controlled by the researchers, age range 18–50 years, and deliberate focus on proximal, rather than temporally distal, risk factors.

### Suicidal Behavior

We found marked differences in suicidal behavior and ideation of the patient groups. Approximately one-third of patients reported an earlier SA, which is in line with earlier Finnish studies of patients with mood disorders (39).The proportion of patients in the MDD subcohort reporting a history of earlier SA was 15%, comparable with the findings of STAR*D and iSpot-D (40, 41) as well as with other Finnish studies (42). Relative to the MDD subcohort, in the BD subcohort previous SAs were twice as prevalent (30%; slightly lower than previous reports of around 40%) (43, 44) and in the BPD subcohort four times more prevalent (60%). Remarkably, over 90% of BD patients with comorbid BPD reported a previous SA, which may indicate cumulative risk, although this finding is in need of replication. Suicide attempts during the index MDE were reported by approximately 9% of patients, which is somewhat lower than in previous Finnish studies of MDD (42) and BD patients, which however, included inpatients. The fact that lifetime, but not recent, prevalence of SA was higher in the BD than in the MDD subcohort is in line with earlier evidence that the increased SA risk of BD patients (relative to MDD patients) is due to their spending more time unwell. Our findings suggest that BD patients have a twofold higher risk of SA than MDD patients, MDD/BPD patients have a twofold higher risk than BD patients, and the risk is cumulative in BD/BPD patients, with the risk being six-fold higher than in MDD patients without BPD.

### Suicidal Ideation

Suicidal ideation is a multifaceted concept, and how much of it is found in a particular sample depends on both how the concept is defined and the tools with which it is measured (45). The CSSRS, which is aimed to capture a wide range of suicidal ideation in a precise manner, indicated some degree of suicidal ideation in the vast majority (87.3%) of our sample. This is a larger proportion than in many previous studies (39) probably indicating a greater sensitivity of the instrument used, although our sampling procedure, which aimed to include sufficient numbers of BPD patients, may also have contributed. Recent suicidal ideation was reported by approximately 60% of BD and MDD patients, comparable to earlier BD (44) and MDD studies (42) As well as being more common, suicidal ideation was also more severe in the BPD subcohort; the median patient in the BPD group reported having had suicidal thoughts with specific methods and some intent to act, whereas median patients in the other subcohorts did not report ever having had suicidal intent. Our findings indicate that BPD/MDE patients have a propensity towards more severe suicidal ideation than other mood disorder patients, and that having had suicidal intent is common in this patient group.

### Risk Factors and Correlations

We examined risk factors for suicidal ideation and attempts, and expectedly found that hopelessness was independently correlated with lifetime risk of previous attempts and severe suicidal ideation, whereas lower age, increased severity of depressive symptoms, and features of BPD were correlated with a higher risk of previous SA. A younger age and more severe features of BPD were independently correlated with a higher risk of both recent attempts and severe ideation, whereas hopelessness and severity of depressive symptoms were related only to the risk of having severe suicidal ideation.

We found that severity of BPD features was independently correlated with suicidal ideation and acts and related to recent severity of suicidal ideation in a dose-dependent manner. To our knowledge, this is the first study to analyze the role of current severity of BPD features, reliably measured by the BPDSI, as a risk factor for suicidal behavior and ideation. According to post-hoc analyses, sensitivity to abandonment, feelings of emptiness, and outbursts of anger or rage may be significantly correlated with significant suicidal ideation or behavior.

In contrast to our hypotheses, we could not confirm earlier reports of a correlation between bipolar mixed states and either suicidal ideation or behavior. Some reasons for this may be our inclusion criterion of current MDE, which may have selected against mixed states, and the lack of precise information regarding the affective states of patients at the time of previous attempts.

### Conclusions

In conclusion, we found marked differences in the prevalence and severity of suicidal ideation and behavior between depressed patients with MDD, BD, or BPD with MDD. The lifetime prevalence of suicidal attempts and other behavior was higher in the BD than in the MDD subcohort, but highest in the BPD/MDD subcohort. BD with comorbid BPD may confer an additive and thus remarkably high risk of suicide attempts. Of all risk factors examined, dimensional measures of BPD severity were most consistently and significantly correlated with the risk for significant ideation and recent SAs. In the light of this study, severity of BPD features is a central modifiable (46, 47) risk factor for suicidal behavior and ideation among mood disorder patients. Thus, measuring this severity reliably (e.g., with the BPDSI) should be implemented in future suicidological research. Clinically, assessment of borderline features may advance evaluation of suicide risk, and their treatment help suicide prevention.

## Data Availability Statement

The datasets for this manuscript are not publicly available, because the research permit of the study does not allow it. Requests to access the datasets should be directed to EI (erkki.isometsa@hus.fi).

## Ethics Statement

The studies involving human participants were reviewed and approved by the Ethics Committee of the Helsinki and Uusimaa Hospital District. The patients/participants provided their written informed consent to participate in this study.

## Author Contributions

JJS, JLS, TR, JE, and EI are part of the research team. EI, JE, JJS, and JLS designed the study. JLS and JJS recruited, interviewed, and assessed the patients. JJS undertook the analysis of this manuscript with TR acting as statistical expert. JJS wrote the first draft of the manuscript. All authors discussed the results and implications and commented on the manuscript. All authors have contributed to and approved the final manuscript.

## Funding

This study has been funded by research grants from the City of Helsinki, the Helsinki and Uusimaa Hospital District, and the Finnish Psychiatric Association.

## Conflict of Interest

The authors declare that the research was conducted in the absence of any commercial or financial relationships that could be construed as a potential conflict of interest.

## References

[1] Cavanagh JT Carson AJ Sharpe M Lawrie SM Psychological autopsy studies of suicide: a systematic review. Psychol Med (2003) 33(3):395–405. 10.1017/S0033291702006943 12701661

[2] Henriksson MM Aro HM Marttunen MJ Heikkinen ME Isometsa ET Kuoppasalmi KI Mental disorders and comorbidity in suicide. Am J Psychiatry (1993) 150(6):935–40. 10.1176/ajp.150.6.935 8494072

[3] Angst J Angst F Gerber-Werder R Gamma A Suicide in 406 mood-disorder patients with and without long-term medication: a 40 to 44 years' follow-up. Arch Suicide Res (2005) 9(3):279–300. 10.1080/13811110590929488 16020171

[4] Paris J Zweig-Frank H A 27-year follow-up of patients with borderline personality disorder. Compr Psychiatry (2001) 42(6):482–7. 10.1053/comp.2001.26271 11704940

[5] Nordentoft M Mortensen PB Pedersen CB Absolute risk of suicide after first hospital contact in mental disorder. Arch Gen Psychiatry (2011) 68(10):1058–64. 10.1001/archgenpsychiatry.2011.113 21969462

[6] Angst J Gamma A Benazzi F Ajdacic V Eich D Rossler W Toward a re-definition of subthreshold bipolarity: epidemiology and proposed criteria for bipolar-II, minor bipolar disorders and hypomania. J Affect Disord (2003) 73(1-2):133–46. 10.1016/S0165-0327(02)00322-1 12507746

[7] Angst J Azorin JM Bowden CL Perugi G Vieta E Gamma A Prevalence and characteristics of undiagnosed bipolar disorders in patients with a major depressive episode: the BRIDGE study. Arch Gen Psychiatry (2011) 68(8):791–8. 10.1001/archgenpsychiatry.2011.87 21810644

[8] Holma KM Haukka J Suominen K Valtonen HM Mantere O Melartin TK Differences in incidence of suicide attempts between bipolar I and II disorders and major depressive disorder. Bipolar Disord (2014) 16(6):652–61. 10.1111/bdi.12195 24636453

[9] Rodante DE Grendas LN Puppo S Vidjen P Portela A Rojas SM Predictors of short- and long-term recurrence of suicidal behavior in borderline personality disorder. Acta Psychiatr Scand (2019) 140(2):158–68. 10.1111/acps.13058 31155713

[10] Klonsky ED May AM Differentiating suicide attempters from suicide ideators: a critical frontier for suicidology research. Suicide Life Threat Behav (2014) 44(1):1–5. 10.1111/sltb.12068 24313594

[11] Baldessarini RJ Tondo L Pinna M Nunez N Vazquez GH Suicidal risk factors in major affective disorders. Br J Psychiatry: J Ment Sci (2019) 215(4): 1–6. 10.1192/bjp.2019.167 31292010

[12] Aaltonen KI Rosenstrom T Baryshnikov I Karpov B Melartin T Suominen K Mediating role of borderline personality disorder traits in the effects of childhood maltreatment on suicidal behaviour among mood disorder patients. Eur Psychiatry: J Assoc Eur Psychiatrists (2017) 44:53–60. 10.1016/j.eurpsy.2017.03.011 28545009

[13] Hawton K Casanas ICC Haw C Saunders K Risk factors for suicide in individuals with depression: a systematic review. J Affect Disord (2013) 147(1-3):17–28. 10.1016/j.jad.2013.01.004 23411024

[14] Schaffer A Isometsa ET Tondo L HM D Turecki G Reis C International Society for Bipolar Disorders Task Force on Suicide: meta-analyses and meta-regression of correlates of suicide attempts and suicide deaths in bipolar disorder. Bipolar Disord (2015) 17(1):1–16. 10.1111/bdi.12271 PMC6296224 25329791

[15] Isometsa E Suicidal behaviour in mood disorders–who, when, and why? Can J Psychiatry (2014) 59(3):120–30. 10.1177/070674371405900303 PMC4079239 24881160

[16] Holma KM Melartin TK Haukka J Holma IA Sokero TP Isometsa ET Incidence and predictors of suicide attempts in DSM-IV major depressive disorder: a five-year prospective study. Am J Psychiatry (2010) 167(7):801–8. 10.1176/appi.ajp.2010.09050627 20478879

[17] Pallaskorpi S Suominen K Ketokivi M Valtonen H Arvilommi P Mantere O Incidence and predictors of suicide attempts in bipolar I and II disorders: A 5-year follow-up study. Bipolar Disord (2017) 19(1):13–22. 10.1111/bdi.12464 28176421

[18] Oquendo MA Currier D Mann JJ Prospective studies of suicidal behavior in major depressive and bipolar disorders: what is the evidence for predictive risk factors? Acta Psychiatr Scand (2006) 114(3):151–8. 10.1111/j.1600-0447.2006.00829.x 16889585

[19] Jylha P Rosenstrom T Mantere O Suominen K Melartin T Vuorilehto M Personality disorders and suicide attempts in unipolar and bipolar mood disorders. J Affect Disord (2016) 190:632–9. 10.1016/j.jad.2015.11.006 26590510

[20] Popovic D Vieta E Azorin JM Angst J Bowden CL Mosolov S Suicide attempts in major depressive episode: evidence from the BRIDGE-II-Mix study. Bipolar Disord (2015) 17(7):795–803. 10.1111/bdi.12338 26415692

[21] Yen S Shea MT Pagano M Sanislow CA Grilo CM McGlashan TH Axis I and axis II disorders as predictors of prospective suicide attempts: findings from the collaborative longitudinal personality disorders study. J Abnormal Psychol (2003) 112(3):375–81. 10.1037/0021-843X.112.3.375 PMC3272767 12943016

[22] Yen S Shea MT Sanislow CA Grilo CM Skodol AE Gunderson JG Borderline personality disorder criteria associated with prospectively observed suicidal behavior. Am J Psychiatry (2004) 161(7):1296–8. 10.1176/appi.ajp.161.7.1296 15229066

[23] Sher L Fisher AM Kelliher CH Penner JD Goodman M Koenigsberg HW Clinical features and psychiatric comorbidities of borderline personality disorder patients with versus without a history of suicide attempt. Psychiatry Res (2016) 246:261–6. 10.1016/j.psychres.2016.10.003 27728869

[24] Soloff PH Chiappetta L Suicidal Behavior and Psychosocial Outcome in Borderline Personality Disorder at 8-Year Follow-Up. J Pers Disord (2017) 31(6):774–89. 10.1521/pedi_2017_31_280 PMC6544464 28263092

[25] Posner K Brown GK Stanley B Brent DA Yershova KV Oquendo MA The Columbia-Suicide Severity Rating Scale: initial validity and internal consistency findings from three multisite studies with adolescents and adults. Am J Psychiatry (2011) 168(12):1266–77. 10.1176/appi.ajp.2011.10111704 PMC3893686 22193671

[26] First MB Spitzer RL Gibbon M Williams JBW Structured Clinical Interview for DSM-IV-TR Axis I Disorders, Research Version, Non-patient Edition. (SCID-I/NP). New York, NY: Biometrics Research, New York State Psychiatric Institute; (2002).

[27] First MB Gibbon M Spitzer RL Williams JBW Benjamin LS Structured Clinical Interview for DSM-IV Axis II Personality Disorders, (SCID-II). Washington, D.C.: American Psychiatric Press, Inc. (1997).

[28] Montgomery SA Asberg M A new depression scale designed to be sensitive to change. Br J Psychiatry (1979) 134:382–9. 10.1192/bjp.134.4.382 444788

[29] Young RC Biggs JT Ziegler VE Meyer DA A rating scale for mania: reliability, validity and sensitivity. Br J Psychiatry (1978) 133:429–35. 10.1192/bjp.133.5.429 728692

[30] Arntz A van den Hoorn M Cornelis J Verheul R van den Bosch WM de Bie AJ Reliability and validity of the borderline personality disorder severity index. J Pers Disord (2003) 17(1):45–59. 10.1521/pedi.17.1.45.24053 12659546

[31] Angst J Gamma A Benazzi F Ajdacic V Eich D Rössler W Toward a re-definition of subthreshold bipolarity: epidemiology and proposed criteria for bipolar-II, minor bipolar disorders and hypomania. Validating Biopolar Spectr (2003) 73(1–2):133–46. 10.1016/S0165-0327(02)00322-1 12507746

[32] Goldman HH Skodol AE Lave TR Revising axis V for DSM-IV: a review of measures of social functioning. Am J Psychiatry (1992) 149(9):1148–56. 10.1176/ajp.149.9.1148 1386964

[33] Beck AT S RA Brown GK Manual for the Beck Depression Inventory-II. San Antonio, TX: Psychological Corporation; (1996).

[34] Kroenke K Spitzer RL Williams JB The PHQ-9: validity of a brief depression severity measure. J Gen Intern Med (2001) 16(9):606–13. 10.1046/j.1525-1497.2001.016009606.x PMC1495268 11556941

[35] Campbell-Sills L Norman SB Craske MG Sullivan G Lang AJ Chavira DA Validation of a brief measure of anxiety-related severity and impairment: the Overall Anxiety Severity and Impairment Scale (OASIS). J Affect Disord (2009) 112(1-3):92–101. 10.1016/j.jad.2008.03.014 18486238 PMC2629402

[36] Zanarini MC Vujanovic AA Parachini EA Boulanger JL Frankenburg FR Hennen J A screening measure for BPD: the McLean Screening Instrument for Borderline Personality Disorder (MSI-BPD). J Pers Disord (2003) 17(6):568–73. 10.1521/pedi.17.6.568.25355 14744082

[37] Blumenthal JA Burg MM Barefoot J Williams RB Haney T Zimet G Social support, type A behavior, and coronary artery disease. Psychosom Med (1987) 49(4):331–40. 10.1097/00006842-198707000-00002 3615762

[38] Beck A BHS, Beck hopelessness scale : manual. San Antonio, Tex: Psychological Corp (1988).

[39] Aaltonen K Naatanen P Heikkinen M Koivisto M Baryshnikov I Karpov B Differences and similarities of risk factors for suicidal ideation and attempts among patients with depressive or bipolar disorders. J Affect Disord (2016) 193:318–30. 10.1016/j.jad.2015.12.033 26774520

[40] Saveanu R Etkin A Duchemin AM Goldstein-Piekarski A Gyurak A Debattista C The international Study to Predict Optimized Treatment in Depression (iSPOT-D): outcomes from the acute phase of antidepressant treatment. J Psychiatr Res (2015) 61:1–12. 10.1016/j.jpsychires.2014.12.018 25586212

[41] Trivedi MH Rush AJ Wisniewski SR Nierenberg AA Warden D Ritz L Evaluation of outcomes with citalopram for depression using measurement-based care in STAR*D: implications for clinical practice. Am J Psychiatry (2006) 163(1):28–40. 10.1176/appi.ajp.163.1.28 16390886

[42] Sokero TP Melartin TK Rytsala HJ Leskela US Lestela-Mielonen PS Isometsa ET Suicidal ideation and attempts among psychiatric patients with major depressive disorder. J Clin Psychiatry (2003) 64(9):1094–100. 10.4088/JCP.v64n0916 14628986

[43] Marangell LB Bauer MS Dennehy EB Wisniewski SR Allen MH Miklowitz DJ Prospective predictors of suicide and suicide attempts in 1,556 patients with bipolar disorders followed for up to 2 years. Bipolar Disord (2006) 8(5 Pt 2):566–75. 10.1111/j.1399-5618.2006.00369.x 17042830

[44] Valtonen H Suominen K Mantere O Leppamaki S Arvilommi P Isometsa ET Suicidal ideation and attempts in bipolar I and II disorders. J Clin Psychiatry (2005) 66(11):1456–62. 10.4088/JCP.v66n1116 16420084

[45] Vuorilehto M Valtonen HM Melartin T Sokero P Suominen K Isometsa ET Method of assessment determines prevalence of suicidal ideation among patients with depression. Eur Psychiatry: J Assoc Eur Psychiatrists (2014) 29(6):338–44. 10.1016/j.eurpsy.2013.08.005 24176645

[46] Vita A De Peri L Sacchetti E Antipsychotics, antidepressants, anticonvulsants, and placebo on the symptom dimensions of borderline personality disorder: a meta-analysis of randomized controlled and open-label trials. J Clin Psychopharmacol (2011) 31:613–24. 10.1097/JCP.0b013e31822c1636 21869691

[47] Cristea IA Gentili C Cotet CD Palomba D Barbui C Cuijpers P Efficacy of psychotherapies for borderline personality disorder: a systematic review and meta-analysis. JAMA Psychiatry (2017) 74:319–28. 10.1001/jamapsychiatry.2016.4287 28249086

